# Variant *brain-derived neurotrophic factor* val66met polymorphism engages memory-associated systems to augment olfaction

**DOI:** 10.1038/s41598-022-24365-5

**Published:** 2022-11-21

**Authors:** Yun-Ting Chao, Tzu-Yi Hong, Ching-Ju Yang, Jen-Chuen Hsieh

**Affiliations:** 1 Institute of Brain Science, National Yang Ming Chiao Tung University, Taipei, Taiwan; 2 Division of Rhinology, Department of Otorhinolaryngology-Head and Neck Surgery, Taipei Veterans General Hospital, Taipei, Taiwan; 3 Integrated Brain Research Unit, Division of Clinical Research, Department of Medical Research, Taipei Veterans General Hospital, Taipei, Taiwan; 4 Department of Biological Science and Technology, College of Biological Science and Technology, National Yang Ming Chiao Tung University, No. 75, Po-Ai Street, Hsinchu, 300 Taiwan; 5 Brain Research Center, National Yang Ming Chiao Tung University, Taipei, Taiwan; 6 Center for Intelligent Drug Systems and Smart Bio-Devices, National Yang Ming Chiao Tung University, Hsinchu, Taiwan

**Keywords:** Genetic association study, Neuroscience, Olfactory system

## Abstract

The neurogenetic basis of variability in human olfactory function remains elusive. This study examined olfactory performance and resting-state functional neuroimaging results from healthy volunteers within the context of the *brain-derived neurotrophic factor* (*BDNF*) val66met polymorphism with the aim of unraveling the genotype-associated intrinsic reorganization of the olfactory network. We found that the presence of the Met allele is associated with better olfactory identification and additional engagement of semantic memory system within the olfactory network, in an allele dosage-dependent manner. This suggests that the Met allele may promote adaptive neural reorganization to augment olfactory capacity.

## Introduction

Brain-derived neurotrophic factor (BDNF) is the most abundant neurotrophin in the brain, playing an important role in cognitive function^1^, memory^2^, and olfaction^3^. In the context of olfaction, BDNF can facilitate neurogenesis in olfactory bulb^4^ and olfactory neuroepithelium^5^, while down-regulated BDNF is associated with apoptotic cell death in olfactory bulb^6^. An interference of BDNF signaling may impede the ability of mice to distinguish odors^7^, and up-regulation of BDNF mRNA in the neuroepithelium is associated with restoration of olfactory function after olfactory training^8^. Collectively, BDNF is a key element for adaptive modulation of the olfactory system at both cellular and behavioral levels.

The *BDNF* val66met polymorphism (rs6265) indicates the substitution of methionine [Met] for valine [Val] at codon 66. The variant Met allele has been shown to interfere with intracellular BDNF-trafficking and decreases activity-dependent BDNF secretion^9,10^. Variant Met allele may deleteriously affect activity-dependent BDNF secretion, and subsequently, the tyrosine receptor kinase B signaling, on which the survival of neuroblasts in olfactory bulbs hinges. The disruptive neurogenesis in olfactory bulbs may lead to impaired olfactory function, particularly discrimination^11^. However, whether the aforementioned findings in the limited basic experimental studies can be translated directly to clinical settings remains relatively unexplored. In fact, several clinical observational reports have presented findings contradictory to the theory as grounded on the basic studies. In one study, otherwise healthy Met carriers presented functional deficits in odor detection threshold, discrimination, and identification^12^. Conversely, other studies counterintuitively reported that the Met allele decelerates age-related olfactory decline late in life^13^ and mediates the recovery of olfactory function in female athletes following concussion^14^. Behavioral studies have suggested that Met carriers may perform better in tasks related to learning and memory^15,16^, and better memory can be one adaptive mechanism that underlies the beneficial olfactory outcome of the Met-carrier aged people and the concussed Met-carrier female athlete. It is highly plausible that memory-associated neural systems, e.g., parahippocampus/posterior cingulate cortex (PCC) subserving cross-modal retrieval and recognition/identification of odor memories^17–19^ and hippocampus/retrosplenial cortex (RSC) subserving long term and autobiographic memories^20–22^, can be further engaged as compensatory and organizational neural mechanisms in patients with defective olfactory performance. Anyhow, researchers have yet to determine whether the presence of the Met allele is associated with neural organization, or whether this neural plasticity has detrimental or protective effects on olfactory capacity.

In the current study, we investigated the genetic attributes of brain organization using functional connectivity (FC) analysis of resting-state functional MRI (rs-fMRI) to unveil the intrinsic circuitry of olfactory network (ON). Resting-state neural activity, whether based on inherent neural connections or adaptive rewiring of the neural networks, is prone to genetic modulation^23,24^. We aimed to clarify whether *BDNF* val66met polymorphisms are associated with different neural endophenotypes, which in turn can be associated with different olfactory performances. Our work may provide a better understanding of the discrepancy among animal and human olfactory studies, and sheds light on the hitherto unexplored neural plasticity of *BDNF* genotype-laden brain organizations.

## Results

### Met allele predicts better olfactory performance

**Figure 1:**
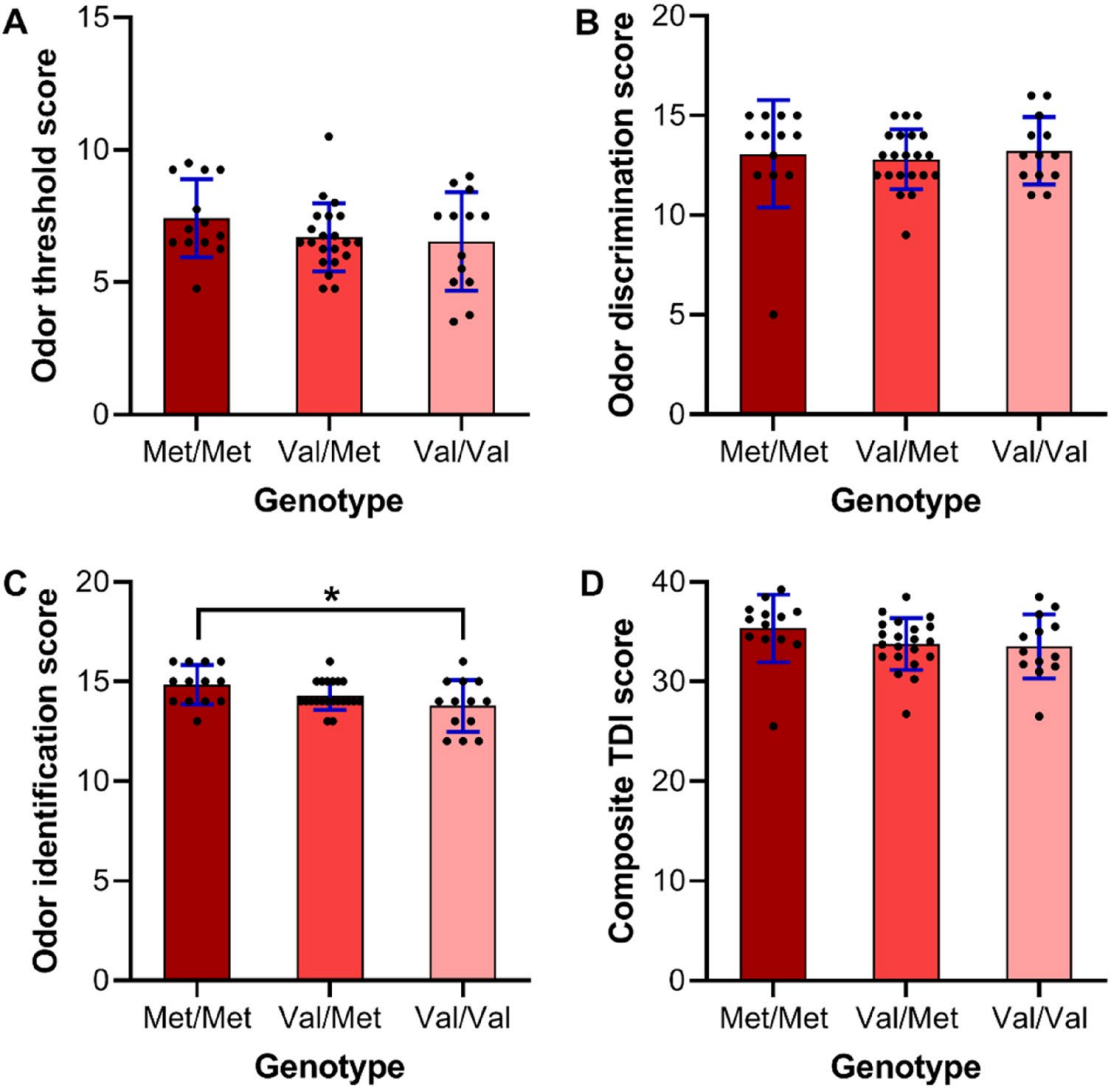
Differences in olfactory performance (by Sniffin’ Sticks test) among the three genotype groups in the olfaction cohort (n = 47). (**A**,**B**,**D**) No differences were noted in terms of odor detection threshold, discrimination, or composite TDI score. (**C**) The Met/Met group out-performed the Val/Val group in odor identification (post hoc Bonferroni test, *P = 0.023). A Met-allele dosage effect was observed (linear regression, *r* = 0.45, beta = 0.61, *t* = 3.02, *P* = 0.004, Supplementary Table 2). The bar graphs show the means and standard deviations of olfactory scores, and the scatter plots demonstrate all data points.

We first examined the relationship between the *BDNF* val66met polymorphism and olfactory performance. A total of 47 healthy subjects (termed as olfaction cohort, 30 females and 17 males; mean age 24.5 ± 2.9 years; Taiwanese ethnicity) from a cohort previously assembled for a *Neuroimaging Program of Primary Dysmenorrhea* (*PDM*) (termed as PDM cohort) were included in the sample. That research program oversaw the genotyping of the *BDNF* val66met polymorphism, measurement of gonadal hormone levels, and acquisition of multimodal neuroimaging including rs-fMRI and structural MRI^25–27^. Since the frequency of Met allele in *BDNF* val66met polymorphism is higher in Asian population (43.6%) than that in Western one (19.9%)^28^, we take the advantage of this preponderant Met allele distribution (Met allele: 47.9% in the current study) and look into the subtle effects contributed by the Met allele. Subjects in the olfaction cohort were grouped according to genotype: Met/Met (n = 13), Val/Val (n = 13), and Val/Met (n = 21). Olfactory ability (odor detection threshold, odor discrimination, and odor identification) was assessed using the Sniffin’ Sticks test^29,30^ (Supplementary Table 1). We observed significant between-group differences (*P* = 0.027, one-way ANOVA) attributable mainly to Met/Met vs. Val/Val (*P* = 0.023, post hoc Bonferroni test) and Met allele dosage-dependent effects (r = 0.45, beta = 0.61, t = 3.02, *P* = 0.004, linear regression; Supplementary Table 2) only in the odor identification subtest (Fig. 1). No between-group differences were observed in terms of sinonasal symptoms, depression, or anxiety. Since odor identification mandates higher-order cognitive processing (e.g., executive function and semantic memory)^31,32^, it is possible that the Met carriers may better recognize odors with the advantage of implicit access of conceptual and semantic contextual information as an adaptive and evolving process. Further behavioral studies are needed to address this speculation.

### Met allele incorporates memory and multi-sensory systems in the olfactory network

**Figure 2:**
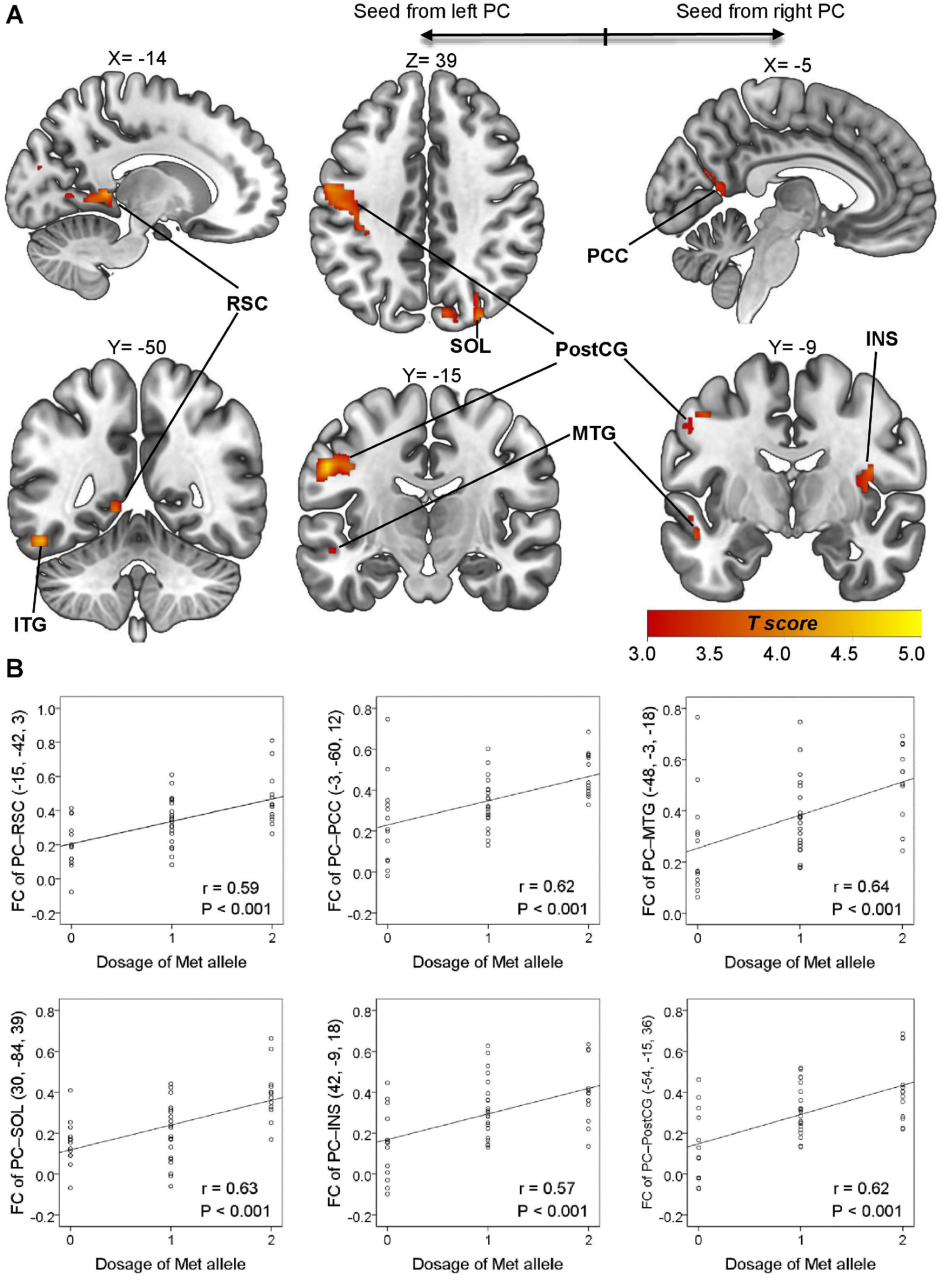
Representations of *BDNF* gene-informed piriform cortex (PC)-seeded olfactory network (olfaction cohort, n = 47). (**A**) The between-group comparison of olfactory networks in the Met/Met and Val/Val group exhibited stronger links to the semantic (middle/inferior temporal gyrus, MTG/ITG) and autobiographical (retrosplenial cortex, RSC; posterior cingulate cortex, PCC) memory areas, visual cortex (superior occipital lobule, SOL), gustatory area (insula, INS) and somatosensory area (postcentral gyrus, PostCG). (**B**) Met-allele dosage effect on the PC-seeded functional connectivity (FC). Note that in the linear regression model, Met/Met was defined as “2”, Val/Met as “1”, and Val/Val as “0”. The peak coordinates and the linear regression results for the Met-allele dosage effect can be found in Table 1.

Next, we examined the link between genotype and the neural organization of ON in the olfaction cohort. Local intrinsic networks are usually organized into correspondent stimulus- or task-induced cortical representations^33^; therefore, we defined bilateral piriform cortices as anatomical ON seed areas centered at MNI coordinates (− 22, 0, − 14) and (22, 2, − 12), based on a meta-analysis of olfactory activation fMRI studies^34^. We performed whole-brain seed-based analysis of rs-fMRI to identify brain regions exhibiting genotypic differences in intrinsic connectivity for olfactory perception. Age and gonadal hormonal levels (estrogen, progesterone, and testosterone) were regressed out as nuisances. Between-group differences in ON connectivity were observed only between Met/Met vs. Val/Val groups. No significant finding was observed in other pairwise comparisons (i.e. Met/Met vs. Val/Met or Val/Met vs. Val/Val). The ON in the Met/Met group presented an additional spatial dimension spanning the inferior temporal gyrus (ITG, BA20), middle temporal gyrus (MTG, BA21), PCC (BA23) and RSC (BA30; Fig. 2A), indicating the engagement of semantic memory system to augment odor identification^35^. In addition, the ON in Met/Met group was shown to engage multiple sensory systems, including the visual (superior occipital lobule, SOL, BA19), somatosensory (postcentral gyrus, PostCG, BA3/1/2), and gustatory (insula, INS, BA13) areas (Fig. 2A). The cross-modal incorporation of contextual information associated with previous sensory experience can help to identify specific odors through the recollection of stored images^31^. Note that the FCs identified in this analysis exhibited a dosage-dependent effect of the Met allele (Fig. 2B and Table 1), where the odor-identification performance shows a cohesive trend with the Met-allele dosage-dependent neural wiring (Fig. 1C). However, the correlations between FCs of ON and odor-identification performance did not achieve statistical significance. To sum up, olfactory performance and brain imaging findings both strongly suggest that the superior odor-identification performance of Met/Met subjects can be attributed to the engagement of semantic memory and multiple sensory cortices within ON.

**Table 1:** Peak coordinates in comparison of olfactory networks in Met/Met and Val/Val groups within the olfaction cohort (n = 47).

Seeds	MNI			T	Peak level	Cluster	Cluster level	BA	Anatomic label	Linear regression for Met-allele dosage effect				
	x	y	z		*P*_*uncorr*_		*P*_*FWE-corr*_			Cohen's f^2^	*r*	beta	*t*	*P*
PC (left) (− 22, 0, − 14)	− 15	− 42	3	4.83	< 0.001	454	< 0.001	30	Retrosplenial cortex (L)	0.55	0.59	0.14	4.56	< 0.001
	− 54	− 15	36	4.81	< 0.001	880	< 0.001	3/1/2	Postcentral gyrus (L)	0.62	0.62	0.15	4.63	< 0.001
	− 51	− 51	− 18	4.50	< 0.001	237	0.003	20	Inferior temporal gyrus (L)	0.60	0.61	0.17	4.47	< 0.001
	30	− 84	39	4.12	< 0.001	307	0.001	19	Superior occipital lobule (R)	0.67	0.63	0.14	5.09	< 0.001
PC (right) (22, 2, − 12)	42	− 9	18	4.10	< 0.001	161	0.017	13	Insula (R)	0.48	0.57	0.13	4.04	< 0.001
	− 45	− 9	48	3.98	< 0.001	198	0.005	3/1/2	Postcentral gyrus (L)	0.54	0.59	0.13	4.28	< 0.001
	− 48	− 3	− 18	3.98	< 0.001	154	0.020	21	Middle temporal gyrus (L)	0.70	0.64	0.15	4.86	< 0.001
	− 3	− 60	12	3.67	< 0.001	143	0.021	23	Posterior cingulate cortex (L)	0.62	0.62	0.14	4.63	< 0.001

*PC* piriform cortex, *SMA* supplementary motor area, *BA* Brodmann area, *L* left, *R* right. Significance in linear regression was defined as *P* = 0.006. (0.05/8, the number of brain areas examined = 8).

### Met allele exhibits the dosage-dependent effect on memory system engagement in the olfactory network

**Figure 3:**
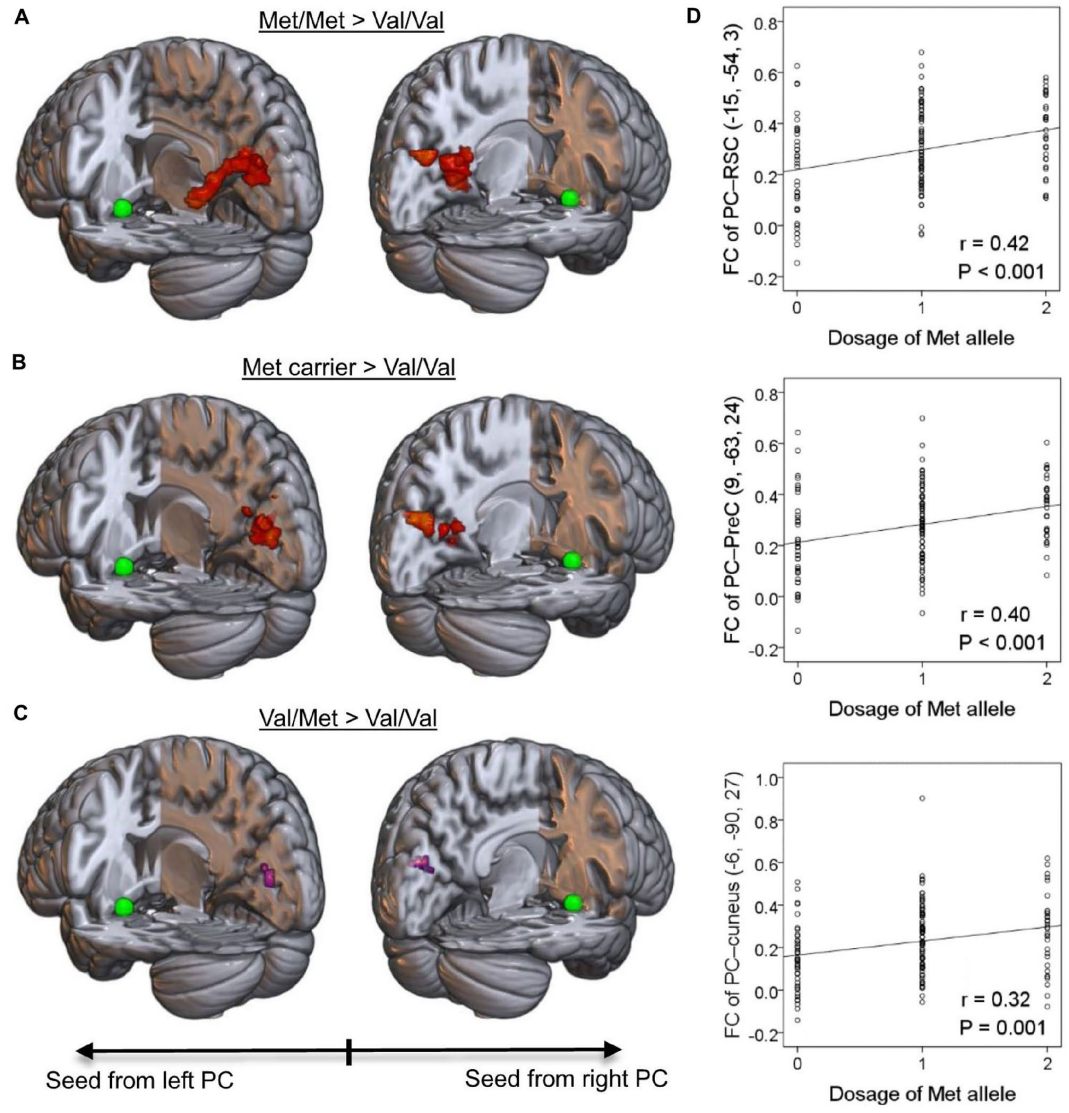
*BDNF* gene-informed piriform cortex (PC, in the green sphere)-seeded olfactory network (PDM cohort n = 145). (**A**) The between-group comparison of olfactory networks in the Met/Met and Val/Val groups exhibited hyper-connectivity to a confluent cluster including the retrosplenial cortex (RSC), posterior cingulate cortex (PCC), precuneus and cuneus (*P*_*FWE-corr.*_ < 0.001, in red). (**B**) The between-group comparison of olfactory networks among the Met carriers and Val/Val groups converged to the RSC and cuneus (*P*_*FWE-corr.*_ < 0.001). (**C**) The between-group comparison of olfactory networks in the Val/Met and Val/Val groups displayed sub-significant clusters in the cuneus (*P*_*FWE-corr.*_ = 0.471 and 0.464 as seeded from the left and right PC respectively, in violet). (**D**) Met-allele dosage effects were observed in all aforementioned PC-seeded neurodynamics. The peak coordinates and linear regression results for the Met-allele dosage effect can be found in Supplementary Table 3.

We then sought to verify whether the observed genotype-related engagement of memory/multi-sensory systems in the ON was generalizable to the overall PDM cohort as a plausible representation of the population. We conducted the same (as olfaction cohort) image processing procedure on the PDM cohort (145 healthy subjects, including 119 females and 26 males; mean age: 23.6 ± 2.6 years old). A comparison of the ON in Met/Met (n = 32) and Val/Val groups (n = 40) revealed that increased intrinsic connectivity in the Met/Met group confluently involved the RSC, PCC, precuneus and cuneus (Fig. 3A). Following a comparison of the ON in Met carriers (Met/Met and Val/Met groups combined, n = 105) and Val/Val group (n = 40), the areas of significance converged to the RSC, precuneus and cuneus (Fig. 3B). A comparison of the ON in the Val/Met (n = 73) and Val/Val groups (n = 40) revealed smaller and sub-significant areas in the cuneus (Fig. 3C). Linear regression analysis verified that the Met allele had a dosage-dependent effect on intrinsic network connectivity (Fig. 3D and Supplementary Table 3). These findings indicate that the Met-allele has a dosage-dependent effect on the engagement of the memory system benefitting odor identification. To investigate whether the findings of Met advantage could be ON specific, we further conducted the same analysis on another sensory network (i.e., visual network, VN). Seed-based FC analyses were conducted on the bilateral primary visual cortices (V1) as seeded at MNI coordinates (− 8, − 76, 10) and (7, − 76, 10), respectively^36^. No significant clusters were detected in all pairwise between-group (genotype) comparisons. Thus, the observed Met advantage may be ON specific.

## Discussion

This study unveiled that the Met allele may shape intrinsic connections within the ON, in an allele dosage-dependent manner, and that these effects encompass memory-associated brain areas leading to superior odor-identification performance in healthy subjects. Many identified areas are important neural substrates of ON (INS, RSC, ITG, PostCG)^20^. Some other regions (MTG, RSC, and PCC) subserve semantic memory^35^, critical for the naming of specific odors^37^. They also constitute the posterior component of the default mode network (DMN), which subserves self-referential processing^38^. The multiple sensory systems identified in the olfactory cohort may be indicative of the assimilation of memory process. The PCC/RSC were preponderant among the identified substrates of ON. The neural organization as revealed in Met carriers functionally mirrors the potential engagement of semantic process^37,39^, which can be a possible mechanism of better identification of odors as compared to Val/Val subjects.

We argue that despite an inherent deficiency in the availability of BDNF (in response to rapid depolarization), the Met allele may promote neuroplastic processes, including the gradual formation, fine-tuning, and stabilization involved in sculpting a neural organization capable of augmenting olfactory function^40^. Contrary to the conventional belief that the Met allele impairs cognitive and perceptual function^9^, it appears that the Met allele may produce a more resourceful network configuration capable of augmenting cognitive performance. Note that this may explain the counterintuitive observation in which the Met allele appears to counteract age-related olfactory decline^13^ and prognosticate better olfactory function in concussed female athletes^14^.

Many factors can be attributed for the incoherent findings between the study of Tonacci et al. and our current study. Tonacci et al. combined the homozygous and heterozygous Met-carrier people (due to scarcity of Met/Met genotype, less than 5%, of the recruited participants) for statistical analyses^12^, while we took the advantage of higher allele frequency in the Taiwanese population and were able to decipher the genotype-specific brain organization and respective olfactory performance of *BDNF* val66met polymorphisms. This highlights the importance of the ethnic factor in the context of different genotype distributions in future studies of genetic neuroimaging and behavioral studies^28^. Other attributing factors include different gender and age distributions.

Our additional examination of the bilateral V1-seeded FCs implicated that the observed Met-advantage neural resilience may be olfactory domain-specific. It can be of interest for future studies to examine whether such Met-advantage neuroplasticity can occur in other sensory modalities.

PDM cohort (n = 145) was three times the size of the olfactory cohort (n = 47), thus the analytic results of PDM (e.g., PCC/RSC—regions subserving semantic memory) may yield the most stable and robust neural dynamics (discerning the individual variations). On the contrary, the results of the smaller-sized olfactory cohort (e.g., multiple sensory systems) may be influenced by the co-engagement of other brain regions (or processes) contextualizing the diversity of individual olfactory experiences. Such individual variations can be vulnerable to averaging process.

The genotype–phenotype interactions are not of simple linear dynamics and can be substantially affected by gene–gene or gene–brain interaction. Although the current study did not demonstrate a significant correlation between the odor-identification performance and neural network FCs, it should be borne in mind that the brain imaging changes can be more sensitive to genetic influences than the behavioral presentations^25,41,42^.

In order not to miss subtle and insightful information that can be valuable for further hypothetical construction and scientific exploration, we therefore opted for a relatively liberal uncorrected *P* = 0.005 approach for our study of relatively small sample size. Such an approach has been adopted by neuroimaging society^43^. The engaged brain regions surviving the stringent uncorrected *P* = 0.001 approach would be additionally marked in the Supplementary Table 3.

The limited sample size and uneven gender distribution in the current study precluded the analysis of gender differences. This cross-sectional study was unable to address the commencement or temporal evolution of neurogenetic plasticity. It remains to be determined whether such *BDNF* genotype-specific neural organization is due to inherent gene-imprinting or long-term neuroplastic processes. Nevertheless, our novel findings shed light on the assessment of clinical symptoms and the prediction of outcomes pertaining to olfactory dysfunction in various clinical settings (e.g., head trauma, aging, and post-viral infection) as well as the tailoring of treatment strategies to specific patients.

## Materials and methods

### Participants

The subjects enrolled in this study were healthy control participants recruited from our *Neuroimaging Program of Primary Dysmenorrhea* (*PDM*), a portion of which has previously been published^25,27,44,45^. The survey in that program included *BDNF* val66met genotyping, measurements of gonadal hormone levels, and multimodal neuroimaging studies including rs-fMRI and structural MRI. Participants with physical or neurological disorders and/or MRI contraindications were excluded from the study. We also excluded subjects presenting head motion exceeding 2 mm or 2 degrees during MRI preprocessing. The final PDM cohort consisted of 145 individuals (119 females and 26 males; mean age of 23.62 ± 2.57 years; Taiwanese ethnicity). Genotype distribution in the PDM cohort did not deviate from the Hardy–Weinberg equilibrium (χ^2^ = 0.017, *P* = 0.991), thereby verifying conformity with the distribution of the general population. Genotype information in the current study was used only for grouping purposes. A subset of participants (termed as olfaction cohort, n = 47; 30 females and 17 males; mean age of 24.45 ± 2.94 years) from the PDM cohort was randomly recruited to undergo assessment of olfactory function. Note that we had originally aimed to recruit participants for the olfaction cohort with a balanced distribution of genotypes (i.e. Met/Met:Val/Met:Val/Val = 1:1:1); however, this goal could not be achieved under the conditions imposed by the COVID-19 pandemic. All subjects were right-handed, as confirmed using the Edinburgh Inventory^46^. All experiment procedures were conducted in accordance with the Declaration of Helsinki and were approved by the local ethics committee of Taipei Veterans General Hospital, and informed consent was provided by every participant.

### Olfactory performance

The participants of the olfaction cohort underwent olfactory evaluations using the Sniffin’ Sticks test (Burghart Instruments, Wedel, Germany). The olfactory exam comprised three subtests: odor detection threshold (T), odor discrimination (D), and odor identification (I)^47,48^. The olfactory threshold for n-butanol was assessed using the adaptive staircase procedure with a three-alternative forced-choice presentation. The odor discrimination task employed 16 triplets of sticks, two of which contained the same odorant with the third containing an odd odorant. The sticks were randomly presented to the participants, who were then required to select the odd one. Odor identification involved 16 common odors, which were identified from a list of descriptors (four for each odor)^30^. The scores in each subtest were summed up to create a composite TDI (threshold-discrimination-identification) score. Nasal conditions and emotional states have been shown to influence olfactory performance; therefore, we also had the participants complete the Sino-Nasal Outcome Test (SNOT-22)^49^, Beck Depression Inventory (BDI-II)^50^, and Beck Anxiety Inventory (BAI)^51^ questionnaires. No significant differences were observed between the three genotype groups in terms of SNOT-22, BDI-II, or BAI scores (Supplementary Table 2).

### Genotyping

All of the participants in the PDM cohort were subjected to genotyping^25,27^. Briefly, whole blood was collected in 4 mL EDTA tubes and stored at 4 °C. DNA was extracted using the Puregene kit in accordance with manufacturer guidelines (Gentra Systems, Minneapolis, MN). Commercial TaqMan single-nucleotide polymorphism assays (Applied Biosystems, Foster City, CA) were used for genotyping. Amplification was performed via the polymerase chain reaction. Fluorescence measurements were performed using the ABI HT7900 (Applied Biosystems, Foster City, CA). Allele calling was performed using the SDS 2.2 software package (Applied Biosystems). Two technicians blinded to the subjects’ personal information assigned genotypes independently.

### Serum levels of gonadal hormones

Inter-subject gonadal hormone variation could conceivably affect the resting-state FC^25,52,53^; therefore, individual serum hormone levels were regressed out as covariates of non-interest during image processing. Hormone-related data were obtained from blood samples drawn from females during the peri-ovulatory phase (i.e., free from menstrual stress), and from all participants on the day they underwent MR scanning. The serum concentrations of estradiol, progesterone, and testosterone were measured using a commercialized assay kit (UniCel DxC 800 Synchron Clinical Systems, Beckman Coulter, Inc., Brea, CA).

### MRI data acquisition

All participants in the PDM and olfaction cohorts were scanned using a 3.0 Tesla MR scanner (Magnetom Trio Tim, Siemens, Erlangen, Germany) equipped with a 12-channel head coil. MR images were acquired from female participants during the peri-ovulatory phase. High-resolution T1-weighted 3-dimensional structural images were acquired (MPRAGE; [TR]/[TE] = 2530 ms/3.03 ms; flip angle = 70°; field-of-view = 224 × 256 mm^2^; in-plane matrix size = 224 × 256 × 192; in-plane resolution = 1 mm). Rs-fMRI images were obtained using a T2*-weighted gradient-echo sequence ([TR]/[TE] = 2500 ms/30 ms; flip angle = 90°; field-of-view = 220 × 220 mm^2^; in-plane matrix size = 64 × 64 × 40; in-plane resolution = 3.4 mm, and 200 volumes). Head cushions and earplugs were respectively provided to reduce head motion and noise. Participants remained awake throughout the scanning session in a resting-state (i.e., eyes open, head still without thinking about anything).

### fMRI data preprocessing

Images were preprocessed using the DPARSF 4.5 Toolbox (State Key Laboratory of Cognitive Neuroscience and Learning, Beijing Normal University, China)^54^ in conjunction with Statistical Parametrical Mapping 12 (SPM12, Wellcome Trust Centre for Neuroimaging, London, http://www.fil.ion.ucl.ac.uk/spm) within a Matlab framework (Matlab R2017b, MathsWorks Inc., Natick, MA, USA). All functional images were subjected to slice timing and realignment for head-motion correction. T1-weighted images from each individual were coregistered to the mean functional image and segmented into gray matter, white matter and cerebrospinal fluid (CSF) signals using the DARTEL Toolbox^55^. We then conducted the nonlinear transformation of individual images to the Montreal Neurological Institute (MNI) space. The Friston 24-parameter model was used to minimize confounding effects due to head motion^56^. Other sources of noise were regressed out by removing white matter and CSF signals. Note that we opted not to perform global signal regression, as this is a controversial practice in studies on FC^57^. Functional images were re-sampled to an isotropic voxel size of 3 × 3 × 3 mm^3^ and normalized to a customized DARTEL template. The images were then smoothed using a 3D Gaussian kernel of 6 mm full-width at half-maximum. After removing linear trends from the resulting time series, temporal band-pass filtering (0.01–0.1 Hz) was performed to extract low-frequency oscillations associated with spontaneous neuronal activity^58^.

### Seed-based FC maps

Bilateral piriform cortices with a 5-mm radius were selected as anatomical seed areas in the ON. The seeds for ON were centered at MNI coordinates (− 22, 0, − 14) and (22, 2, − 12), based on a meta-analysis of fMRI-activation during olfactory task performance^34^. Similar seed-based FC analyses were conducted on the bilateral V1 as seeded at MNI coordinates (− 8, − 76, 10) and (7, − 76, 10), respectively^36^, to verify whether the neuroplastic changes could be olfactory domain-specific. Following the extraction of mean time-series activity from each seed region, seed-based FC maps were generated for group analysis. Each FC map at the individual level was then converted into a z-map using Fisher’s r-to-z transformation for second-level group analysis.

### Statistical analysis

One-way analysis of variance (ANOVA) was used for the between-group analysis of behavioral and functional imaging data. The Bonferroni method was used for post hoc behavioral analysis with the level of significance set at *P* < 0.05. In whole-brain imaging analysis, covariates (age as well as estrogen, progesterone, and testosterone levels) were regressed out as nuisances. Post hoc between-group comparison of genotype was also performed in both cohorts. The threshold for significance was first set at an uncorrected voxel level of *P* = 0.005, and then at a second corrected cluster level of *P* = 0.05 to estimate family-wise error (FWE). To test for *BDNF* val66met Met allele dosage-dependent effects, we constructed a linear regression model using the val66met genotype coded according to the number of Met alleles (Met/Met = 2, Val/Met = 1, and Val/Val = 0) as the independent variable, and odor identification scores or FC strength as dependent variables. Age as well as levels of estrogen, progesterone, and testosterone were also set as covariates. Bonferroni correction was used to control for type I error attributable to multiple comparisons; therefore, the level of significance was adjusted according to the number of brain areas examined. Consequently, the significance of Met-allele dosage effects on FC in the olfaction cohort in the second experiment was *P* = 0.006 (0.05/8, Table 1); and that of the PDM cohort in the third experiment was *P* = 0.003 (0.05/17, Supplementary Table 3).

## Supplementary Information

Supplementary Tables.

## Data Availability

The authors declare that the data supporting the findings of this study are available within the paper and its supplementary information files.
