# Supplementary material for: Variant brain-derived neurotrophic factor val66met polymorphism engages memory-associated systems to augment olfaction
Source: Sci Rep. 2022 Nov 21;12:20007. doi: 10.1038/s41598-022-24365-5 (PMC9678911; doi:10.1038/s41598-022-24365-5)
Supplement: Supplementary file 1 — Supplementary Tables. [file 41598_2022_24365_MOESM1_ESM.pdf]

Supplementary materials

Variant *brain-derived neurotrophic factor* val66met polymorphism engages memory-associated systems to augment olfaction

Yun-Ting Chao[1,2,3], Tzu-Yi Hong[1,3], Ching-Ju Yang[1,3], Jen-Chuen Hsieh[1,3,4,5,6]

[1]Institute of Brain Science, National Yang Ming Chiao Tung University, Taipei, Taiwan

[2]Division of Rhinology, Department of Otorhinolaryngology-Head and Neck Surgery, Taipei Veterans General Hospital, Taipei, Taiwan

[3]Integrated Brain Research Unit, Division of Clinical Research, Department of Medical Research, Taipei Veterans General Hospital, Taipei, Taiwan

[4]Department of Biological Science and Technology, College of Biological Science and Technology, National Yang Ming Chiao Tung University, Hsinchu, Taiwan

[5]Brain Research Center, National Yang Ming Chiao Tung University, Taipei, Taiwan

[6]Center for Intelligent Drug Systems and Smart Bio-devices, National Yang Ming Chiao Tung University, Hsinchu Taiwan.

Supplementary table 1 original data on olfactory subtests of each subject

No.	Genotype	T	D	I	TDI
1	Met/Met	9.25	14	14	37.25
2	Val/Met	6.75	15	14	35.75
3	Val/Met	6.5	11	15	32.5
4	Val/Met	8.25	12	15	35.25
5	Val/Met	5.25	11	14	30.25
6	Val/Met	7.5	15	14	36.5
7	Val/Met	4.75	12	14	30.75
8	Val/Met	10.5	14	14	38.5
9	Met/Met	9.25	15	15	39.25
10	Val/Met	8	13	16	37
11	Val/Met	6.5	14	14	34.5
12	Val/Val	7.5	16	15	38.5
13	Val/Met	7	12	15	34
14	Val/Met	6.75	14	14	34.75
15	Val/Val	7.5	13	12	32.5
16	Val/Met	6	15	15	36
17	Met/Met	4.75	14	15	33.75
18	Val/Met	6.25	13	15	34.25
19	Val/Val	9	12	14	35
20	Val/Val	7.5	13	14	34.5
21	Met/Met	7.75	12	16	35.75
22	Met/Met	6.5	15	15	36.5
23	Met/Met	9.5	15	14	38.5
24	Val/Met	5.75	14	14	33.75

No.	Genotype	T	D	I	TDI
25	Val/Val	8.5	11	12	31.5
26	Met/Met	6.5	12	16	34.5
27	Met/Met	6.25	12	16	34.25
28	Met/Met	6.75	15	15	36.75
29	Val/Met	6.5	12	14	32.5
30	Met/Met	9.25	14	13	36.25
31	Met/Met	7.25	13	14	34.25
32	Met/Met	6.5	5	14	25.5
33	Val/Val	5	15	13	33
34	Val/Met	5.75	12	14	31.75
35	Val/Met	6.5	13	13	32.5
36	Val/Met	4.75	9	13	26.75
37	Val/Met	6.25	13	14	33.25
38	Val/Met	7.5	12	14	33.5
39	Val/Val	7.5	14	16	37.5
40	Val/Met	7.5	13	15	35.5
41	Val/Val	8.75	14	14	36.75
42	Val/Val	5	12	15	32
43	Val/Val	3.5	11	12	26.5
44	Val/Val	3.75	13	15	31.75
45	Val/Val	5.5	16	14	35.5
46	Val/Val	6	12	13	31
47	Met/Met	7	14	16	37

T: threshold; D: discrimination; I: identification; TDI: T + D + I

Supplementary Table 2 behavioral factors in the olfaction cohort (n = 47)

Behavioral measurement (mean ±SD)	Genotype			ANOVA		Linear regression for Met-allele dosage effect				
	Met/Met (n=13)	Val/Met (n=21)	Val/Val (n=13)	F	*P*	Cohen's f^2	*r*	beta	*t*	*P*
Detection threshold	7.42 ±1.48	6.69 ±1.29	6.53 ±1.86	1.323	0.277	0.08	0.26	0.45	1.40	0.170
Odor discrimination	13.08 ±2.69	12.81 ±1.50	13.23 ±1.69	0.267	0.818	0.02	0.14	0.03	0.08	0.939
Odor identification	14.84 ±0.99	14.29 ±0.72	13.77 ±1.30	3.967	0.027	0.25	0.45	0.61	3.02	0.004
Composite TDI score	35.35 ±3.40	33.79 ±2.61	33.54 ±3.22	2.419	0.246	0.08	0.27	1.09	1.71	0.095
Sino-nasal Outcome test	7.62 ±10.25	8.62 ±9.45	10.62 ±8.78	0.339	0.714	0.06	0.24	-1.87	-0.94	0.353
Beck depression Inventory	3.15 ±4.91	4.33 ±4.98	3.15 ±2.48	0.413	0.664	0.03	0.18	0.00	0.00	0.999
Beck anxiety inventory	3.08 ±4.61	2.57 ±5.33	2.00 ±2.08	0.19	0.828	0.08	0.27	0.71	0.77	0.446

SD, standard deviation; TDI, threshold + discrimination + identification

Supplementary Table 3 Peak coordinates in comparison of olfactory networks among various genotypes in the PDM cohort (n = 145)

Genotype contrast	Seeds	MNI				Peak level	Cluster level				linear regression for Met-allele dosage effect				
		x	y	z	T	$P_{uncorr.}$	Cluster	$P_{FWE\text{-}corr.}$	BA	Anatomic label	cohen's f^2	*r*	beta	*t*	*P*
**Met/Met > Val/Val**	**PC (left)** (-22, 0, -14)	-15	-45	3	4.08	< 0.001	711*	< 0.001	29	Retrosplenial cortex (L)	0.19	0.40	0.07	3.75	< 0.001
		-6	-54	12	3.98	< 0.001			30	Retrosplenial cortex (L)	0.21	0.41	0.07	3.75	< 0.001
		9	-63	24	3.90	< 0.001			7	Precuneus (R)	0.19	0.40	0.07	3.91	< 0.001
	**PC (right)** (22, 2, -12)	-15	-54	3	4.04	< 0.001	576*	< 0.001	30	Retrosplenial cortex (L)	0.22	0.42	0.07	3.87	< 0.001
		-6	-87	27	3.73	< 0.001			18	Cuneus (L)	0.20	0.41	0.07	3.83	< 0.001
		-3	-69	21	3.64	< 0.001			18	Cuneus (L)	0.26	0.45	0.07	3.81	< 0.001
**Met carrier > Val/Val**	**PC (left)** (-22, 0, -14)	0	-90	18	3.93	< 0.001	403*	< 0.001	18	Cuneus	0.13	0.34	0.06	3.10	0.002
		-9	-96	27	3.54	< 0.001			19	Cuneus (L)	0.10	0.30	0.04	3.15	0.002
		9	-63	24	3.43	< 0.001			7	Precuneus (R)	0.19	0.40	0.07	3.91	< 0.001
	**PC (right)** (22, 2, -12)	-6	-90	27	4.30	< 0.001	404	< 0.001	19	Cunues (L)	0.12	0.32	0.06	3.26	0.001
		-15	-54	3	3.51	< 0.001			30	Retrosplenial cortex (L)	0.22	0.42	0.07	3.87	< 0.001
		12	-78	24	3.47	< 0.001			18	Cuneus (R)	0.19	0.40	0.05	3.21	0.002
**Val/Met > Val/Val**	**PC (left)** (-22, 0, -14)	0	-90	18	3.67	< 0.001	72	0.471	18	Cuneus	0.13	0.34	0.06	3.10	0.002
		-9	-96	24	3.28	0.001			19	Cuneus (L)	0.08	0.27	0.05	2.63	0.010
	**PC (right)** (22, 2, -12)	-6	-90	27	3.84	< 0.001	71	0.464	19	Cuneus (L)	0.12	0.32	0.06	3.26	0.001
		0	-90	21	3.39	< 0.001			18	Cuneus	0.14	0.35	0.05	2.577	0.01
		-18	-93	27	2.89	0.001			19	Cuneus (L)	0.07	0.25	0.04	2.397	0.02

PC, piriform cortex; BA, Brodmann area; L,left; R,right. Significance in linear regression was defined as *P* = 0.003. (0.05/17, the number of brain areas examined=17)

*The asterisks indicate the clusters that can also survive, but to a lesser spatial extent, the more stringent threshold criteria: uncorrected *P* < 0.001.
